# Dietary Sea Buckthorn Pomace Induces Beige Adipocyte Formation in Inguinal White Adipose Tissue in Lambs

**DOI:** 10.3390/ani9040193

**Published:** 2019-04-24

**Authors:** Ting Zhang, Buhao Deng, Ruixin Zhang, Xuze Qin, Jianxin Zhang, Junxing Zhao

**Affiliations:** Department of Animal Sciences and Veterinary Medicine, Shanxi Agricultural University, Taigu 030801, China; tzh3058@163.com (T.Z.); Dengbuhao248@163.com (B.D.); ruixinzhang1719@163.com (R.Z.); XuZeQin799@gmail.com (X.Q.)

**Keywords:** mitochondrial, flavonoid, beige, sheep, AMPK

## Abstract

**Simple Summary:**

Heat production is important for the survival of young animals, especially for those born in a cold winter. During this process, both brown and beige adipose tissues play an important role. Sea buckthorn is a natural source of bioactive compounds and has multiple health benefits. The aim of this study was to determine whether dietary sea buckthorn pomace supplementation could affect beige cell formation in lambs. Here, we found that dietary sea buckthorn pomace could promote beige adipocyte formation, increase mitochondrial numbers, and improve insulin sensitivity in inguinal white adipose tissue. These results will be useful for increasing heat production through modulation of beige cell formation in young lambs.

**Abstract:**

The sea buckthorn contains substantial amounts of bioactive compounds. The objective of this study was to investigate the effects of dietary sea buckthorn pomace (SBP) on sheep beige adipocyte formation. A total of thirty lambs were equally divided into three groups and fed with diets containing different levels of SBP: 0% SBP (Control), 7.8% SBP (7.8SBP), and 16.0% SBP (16SBP). The results showed that dietary SBP affected inguinal adipocytes’ size distribution, and increased both UCP1 protein content (*p* < 0.05) and mitochondrial numbers (*p* < 0.05). mRNA expression of peroxisome proliferator-activated receptor gamma coactivator 1α (PGC-1α), nuclear respiratory factor 1, and mitochondrial transcription factor A were increased when animals were subjected to 16% SBP (*p* < 0.05). Supplementation with 16% SBP increased CCAAT/enhancer-binding protein β content (*p* < 0.05) and PR domain containing 16 mRNA abundance (*p* < 0.05). Consistently, inguinal white adipose tissue (iWAT) from the 16SBP group exhibited increased insulin sensitivity, which was associated with elevated glucose transporter 4 abundance (*p* < 0.05). Importantly, AMP-activated protein kinase (AMPK) was activated in the 16SBP group (*p* < 0.05). Collectively, these results suggest that dietary SBP promotes iWAT browning in lambs, which might be through the activation of the AMPK–PGC-1α–UCP1 signaling pathway.

## 1. Introduction

Sea buckthorn (*Hippophae rhamnoides L.*) is a deciduous shrub, naturally distributed between the Atlantic coast of Europe and Northwestern Mongolia and China, and its medicinal and therapeutic potential have been recognized for centuries [1]. Previous studies showed that juices, jams, and oil derived from sea buckthorn have a range of beneficial anti-inflammatory, anticancer, antioxidant, and anti-atherosclerotic effects, which have been attributed to the presence of phenolics, flavonoids, vitamins, minerals, amino acids, fatty acids, and phytosterols [2]. Sea buckthorn pomace (SBP) is a byproduct produced during sea buckthorn juice extraction, and contains substantial amounts of valuable natural bioactive compounds [3]. Thus, the application of SBP as a feed supplement in the husbandry industry provides promising possibilities to improve animal health. Kaushal et al. pointed out that the fruits and seeds of sea buckthorn were suitable for animal feed [4]. In pigs, dietary SBP supplementation did not affect growth performance, slaughter quality traits, or meat quality, but influenced the fatty acid profile of the longissimus muscle [5]. In broiler chickens, dietary supplementation of sea buckthorn flavones has been demonstrated to affect growth performance and fat deposition by regulating lipid metabolism [6]. Interestingly, supplementation of SBP to laying hens showed beneficial effects on the total number of laid eggs and the egg yolk color [7].

Brown adipose tissue (BAT) is the major tissue responsible for non-shivering thermogenesis, which can enhance energy expenditure, due to their high content of mitochondria and uncoupling protein-1 (UCP1). In young lambs, heat produced by BAT accounts for about half of the heat needed by newborns, and the remainder is produced by muscle thermogenesis [8]. Although the BAT-produced heat is essential for the survival of neonatal mammals in cold environments, it disappears rapidly during the postnatal period and, in adults, is difficult to identify [9]. Recently, an inducible form of thermogenic adipocytes, named beige adipocytes (or brite adipocytes) and mainly residing within white adipose tissue, was identified [10]. Similar to brown adipocytes, beige adipocytes possess abundant cristae-dense mitochondria that express UCP1, and thus, can also generate heat under external stimuli [11]. Whether components in sea buckthorn affect beige adipocytes’ development and maintenance in lambs remains unclear. The aim of the current study was to investigate the effects of dietary SBP supplementation on beige adipocyte formation and mitochondrial biogenesis in inguinal white adipose tissue (iWAT) in ram lambs.

## 2. Materials and Methods 

### 2.1. Care and Use of Animals 

All animal procedures were carried out in accordance with the Guidelines for the Care and Use of Laboratory Animals prepared by the Institutional Animal Care and Use Committee of Shanxi Agricultural University. A total of thirty ram lambs (22.2 ± 0.92 kg) were randomly selected and equally assigned to three groups, in a completely randomized design. Lambs (housed in individual stalls) were fed with diets containing different levels of SBP (on a DM (Dry mass) basis): 0% SBP (control group), 7.8% by DM SBP (7.8SBP) and 16.0% by DM SBP (16SBP). The total flavonoid content of the extracts was determined spectrophotometrically as previously described [12]. Ingredients and nutrient contents of experimental diets are shown in Table 1. All animals were fed an experimental diet for 10 days ad libitum for adaptation, and the feeding trial was then initiated and lasted for 80 days before sampling. At the end of the trial, all animals were anesthetized and exsanguinated, and the iWAT tissues were sampled. One piece was rinsed in phosphate-buffered solution (PBS) and fixed in 4% paraformaldehyde (PFA) for paraffin embedding, and the remainder were snap-frozen in liquid nitrogen for further biochemical analysis.

### 2.2. Hematoxylin and Eosin Staining and Adipocyte Diameter Analysis

The hematoxylin and eosin staining (H&E) staining was performed according to a previously published protocol with slight modifications [13]. Briefly, samples of iWAT fixed by 4% PFA (pH 7.4) were serially dehydrated in ethanol and xylene and embedded in paraffin. Each block was sectioned at a thickness of 7 μm by microtome (Leica, Wetzlar, Germany), and was dewaxed and rehydrated serially by incubation with xylene and different concentrations of ethanol. After that, the sections were subjected to H&E staining. Histological examination and imaging were done under microscope (BX53F, Olympus, Tokyo, Japan) at a 100× magnification, and 10 fields from each sample were selected randomly for observation. The diameter was determined using the Image J software (NIH, Bethesda, MD, USA). 

### 2.3. Real-Time Quantitative PCR (qRT-PCR) Analysis

The qRT-PCR was performed according to a previously described method [13]. Briefly, all RNA in the iWAT sample was extracted using the Trizol reagent (Sigma, Saint Louis, MO, USA), and the concentration and integrity of the RNA samples was determined by a NanoDrop instrument (ND-2000, Nanodrop Instruments, DE, USA). cDNAs were synthesized using a reverse transcription kit (TAKARA Co., Ltd., Dalian, China), and qRT-PCR was performed using the CFX RT-PCR detection system (Bio-Rad, Hercules, CA, USA). The PCR cycle parameters were as follows: 36 3-step cycles at 95 °C, 20 s; 55 °C, 20 s; and 72 °C, 20 s. After amplification, a melting curve (0.01 °C/s) was carried out for confirmation of the product’s purity. The 2^−△△Ct^ method was used to analyze the relative changes in the target genes’ expression by normalizing to the *PRL13* content. The primer sets used are listed in Table 2.

### 2.4. Mitochondrial DNA Copy Number Determination

The relative mitochondrial DNA (mtDNA) copy number was determined as previously described [14]. Briefly, all DNA was extracted from the iWAT samples using the traditional phenol–chloroform DNA extraction methods. The mtDNA copy number was measured by amplification of the mitochondrial D-Loop versus the nuclear *GAPDH* gene using a CFX RT-PCR detection system (Bio-Rad, Hercules, CA, USA). The difference in CT values, between the D-Loop and *GAPDH*, was used for calculation of the relative abundance of the mitochondrial genome. 

### 2.5. Western Blotting Analysis

Western blotting analysis was performed by following the established protocol in our lab [15]. Briefly, the iWAT samples (100 mg) were homogenized in 500 μL ice-cold lysis buffer and the homogenates were centrifuged for 15 min at 12,000× *g* and 4 °C for soluble protein separation. Then, the supernatant was mixed with an equal amount of the sample-loading buffer and boiled for 3 min before use. The samples were subjected to SDS-PAGE, and immunoreactive proteins in the membranes were scanned and analyzed using an Odyssey Infrared Imaging System (LI-COR Biosciences, Lincoln, NE, USA). The target band density was normalized according to the β-actin content. 

Antibodies against UCP1 (bs-1925R), CCAAT/enhancer-binding protein β (C/EBPβ, bs-1396R), peroxisome proliferator-activated receptor γ (PPARγ, bs-4590R), p-Akt (33280M), insulin receptor substrate 1 (IRS1, bs-0172R), p-IRS1 (bs-2736R), p-p38 (bs-2210R), and glucose transporter 4 (Glut4, bs-0384R) were obtained from Biosynthesis Biotechnology Co., Ltd. (Beijing, China). AMP-activated protein kinase α (AMPKα, no. 2532), p-AMPKα (no. 2535), Akt (no. 9272), p-38 MAPK (no. 9212), and β-actin (no. 4970) were obtained from Cell Signaling (Danvers, MA, USA). Goat anti-mouse secondary antibody (926-68070) and anti-rabbit secondary antibody (926-32211) were obtained from LI-COR Biosciences (Lincoln, NE, USA).

### 2.6. Statistical Analysis

Statistical analysis was performed using the Graphpad Prism 6 software package (Monrovia, CA, USA). Ten lambs were raised individually in each group and each animal was considered as an experimental unit. Data were expressed as mean ± standard error of mean (SEM) and analyzed using one-way analysis of variance (ANOVA) followed by Tukey’s honestly significant difference (HSD) test. *p* < 0.05 was considered to indicate statistical significance for all data. 

## 3. Results

### 3.1. Dietary SBP Supplementation Affects Browning of iWAT

**Figure 1:**
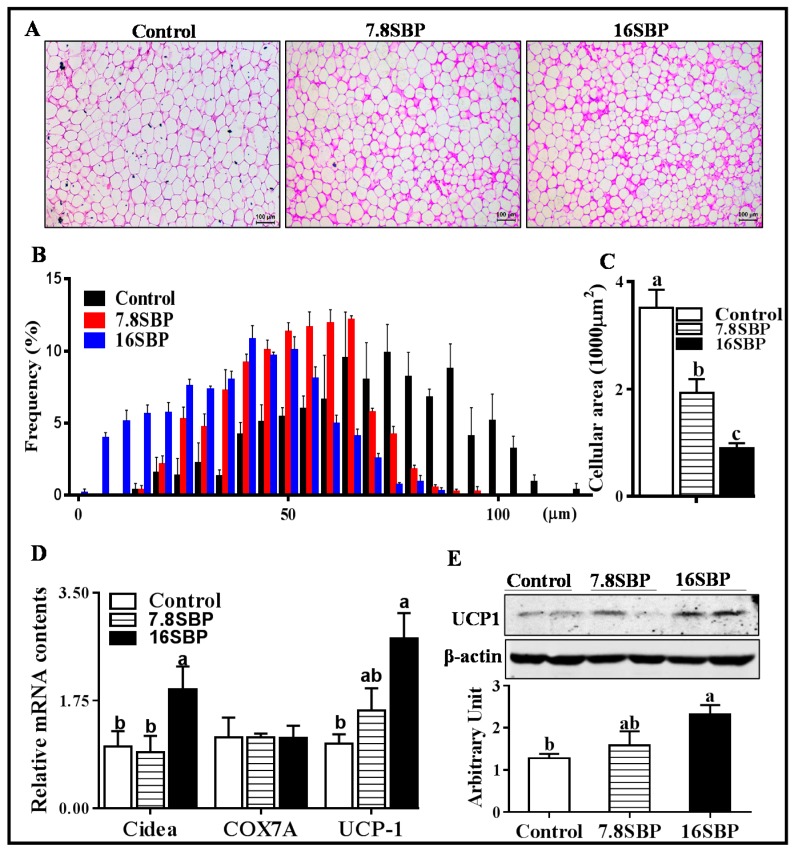
Dietary SBP affects the browning of iWAT of lambs fed with the control (□), 7.8% SBP (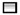) and 16% SBP diet (■). (**A**) H&E staining of iWAT, scar bar stands for 100 μm. (**B**) Adipocyte size distribution. (**C**) Average cellular cross-sectional areas. (**D**) mRNA expression of *Cidea*, *COX7A* and *UCP1*. (**E**) UCP1 protein content analyzed by western blotting. (Mean ± SEM; n = 10; different letters mean significant difference).

As shown in Figure 1A,B, dietary SBP supplementation affected iWAT adipocyte size distribution. Compared with the control group, lambs in both the 7.8SBP and 16SBP groups exhibited a decreased average cross-sectional area of iWAT (Figure 1C, *p* < 0.01). The mRNA expression of cell death-inducing DNA fragmentation factor alpha-like effector A (*Cidea*) was increased when lambs were fed the 16% SBP diet (*p* < 0.05), and no difference was observed between the control and 7.8% SBP supplementation groups (Figure 1D). There was no difference in cytochrome C oxidase subunit 7A (*COX7A*) mRNA content among the three groups (Figure 1D). Although 7.8% SBP supplementation did not alter *UCP1* mRNA expression, lambs fed the 16% SBP diet showed greater *UCP1* mRNA content (*p* < 0.05, Figure 1D). As expected, lambs fed the 16% SBP had greater UCP1 protein content than lambs in the control group (*p* < 0.05, Figure 1E).

### 3.2. Effect of Dietary SBP Supplementation on Mitochondrial Numbers

**Figure 2:**
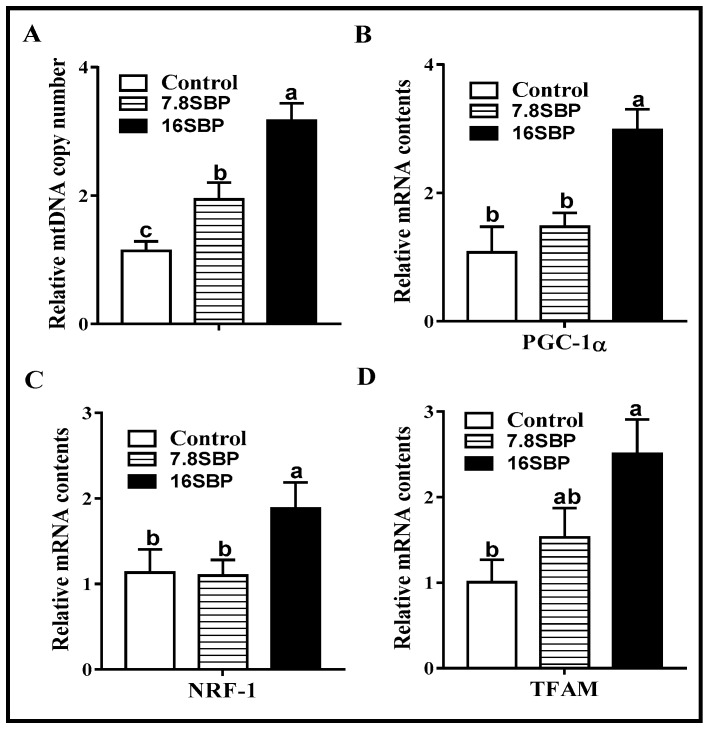
Mitochondrial numbers were changed among different groups. (**A**) Relative mtDNA copy number. (**B**–**D**) mRNA expression of *PGC-1α*, *NRF-1* and *TFAM*. (Mean ± SEM; n = 10; different letters mean significant difference).

Increased mitochondrial numbers were observed in iWAT from both 7.8% and 16% SBP supplementation lambs compared to that from control lambs (Figure 2A, 16SBP > 7.8SBP > control, *p* < 0.05). Both peroxisome proliferator-activated receptor gamma coactivator 1α (*PGC-1α*, Figure 2B) and nuclear respiratory factor 1 (*NRF-1*, Figure 2C) mRNA contents were increased when the animals were subjected to 16% SBP (*p* < 0.05), and no difference was observed between the control and 7.8SBP groups. Compared with the control group, supplementation of 16% SBP in the lamb’s diet increased mitochondrial transcription factor A (*TFAM*) mRNA abundance (Figure 2D, *p* < 0.05).

### 3.3. Dietary SBP Supplementation Affects Beige Adipogenic Transcription Factors Abundance

**Figure 3:**
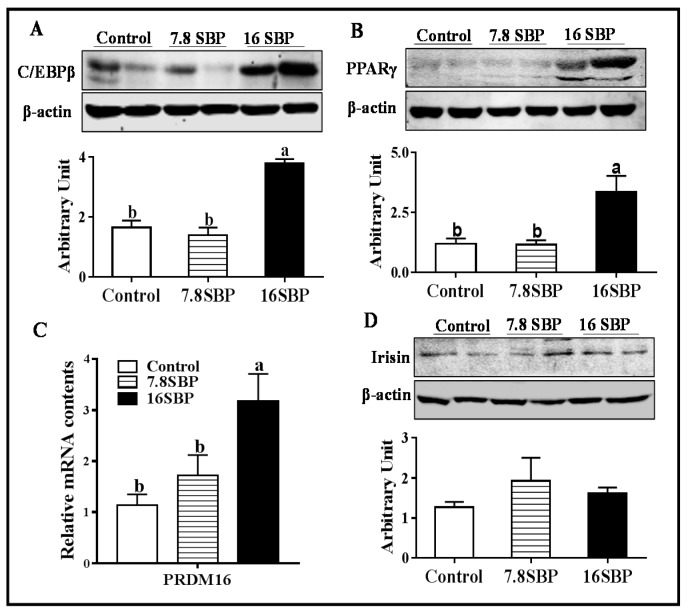
Beige-related transcription factor contents among different groups. (**A**) C/EBPβ protein content. (**B**) PPARγ protein content. (**C**) *PRDM16* mRNA abundance. (**D**) Irisin protein abundance. (Mean ± SEM; n = 10; different letters mean significant difference).

As shown in Figure 3A, 16% SBP supplementation significantly increased C/EBPβ content (*p* < 0.05), and no difference was observed between control and 7.8SBP group. The PPARγ expression pattern exhibited a similar trend to that of C/EBPβ (Figure 3B, *p* < 0.05). Moreover, lambs in the 16SBP group showed an increased PR domain containing 16 (*PRDM16*) mRNA abundance (Figure 3C, *p* < 0.05). In addition, dietary SBP supplementation did not affect irisin abundance (Figure 3D).

### 3.4. Dietary SBP Supplementation Enhanced Insulin Sensitivity in iWAT

**Figure 4:**
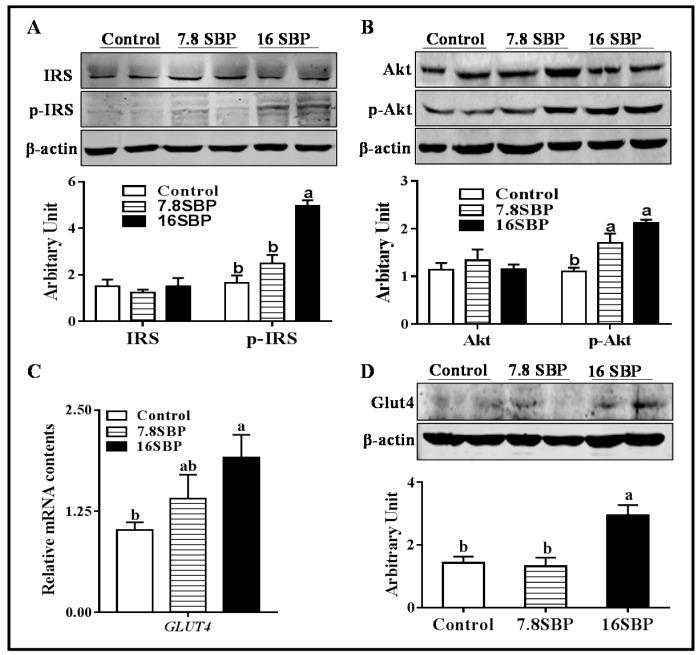
Dietary SBP supplementation enhanced insulin sensitivity. (**A**) Total IRS1 and p-IRS1 protein contents. (**B**) Total Akt and p-Akt protein contents. (**C**) *Glut4* mRNA abundance. (**D**) Glut4 protein content. (Mean ± SEM; n = 10; different letters mean significant difference).

Although total IRS1 protein content was not affected by SBP supplementation, 16% SBP supplementation effectively increased phospho-IRS1 protein abundance (Figure 4A, *p* < 0.05). Meanwhile, lambs subjected to both 7.8% and 16% SBP exhibited greater phospho-Akt levels than lambs in control group (Figure 4B, *p* < 0.05). *Glut4* mRNA abundance was increased in 16SBP group (Figure 4C, *p* < 0.05), and as expected, an elevated Glut4 protein content was observed in the same group (Figure 4D, *p* < 0.05).

### 3.5. Effect of Dietary SBP Supplementation on p38 MAPK and AMPK Activity

**Figure 5:**
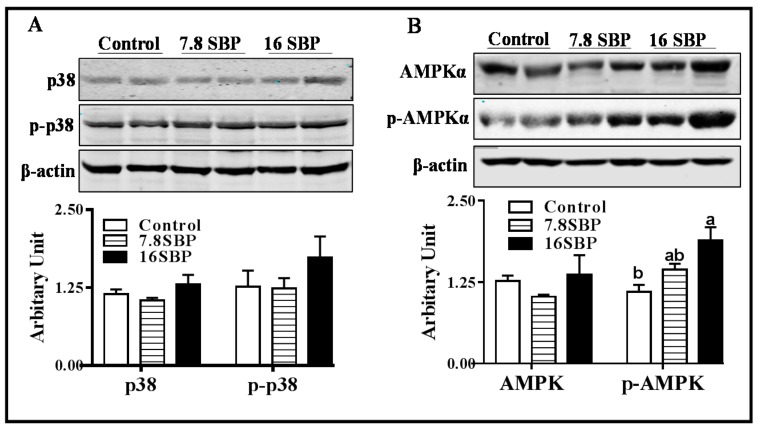
p38 MAPK and AMPK activity among different groups. (**A**) Measurements of p38 and phospho-p38 protein abundances. (**B**) AMPK and phosphor-AMPK protein abundances. (Mean ± SEM; n = 10; different letters mean significant difference).

As shown in Figure 5A, dietary SBP supplementation did not alter either p38 or phospho-p38 protein contents in iWAT among different groups. Although total AMPK protein abundance was not altered by the dietary SBP supplementation, the phospho-AMPK protein content was elevated when lambs were subjected to the 16% SBP-containing diet, and no difference was observed between the control and 7.8SBP groups (Figure 5B, *p* < 0.05).

## 4. Discussion

Previous studies demonstrated that natural flavonoid derivatives effectively activate BAT development and beige fat formation [16]. Notably, flavonoids are one of the major biologically active substances found abundantly in sea buckthorn [17]; thus, we tested whether dietary SBP supplementation affected the browning of white adipose tissue in lambs. We dissected iWAT after lambs were slaughtered, and H&E staining data clearly showed that dietary SBP supplementation led to reduced cellular area. To explore whether beige adipocyte numbers were altered, we analyzed key genes involved in beige adipogenesis. *Cidea* has been employed to evaluate the induction of beige adipocytes in white adipose tissue. The increased mRNA expression of *Cidea* in the 16SBP group suggested an increased number of beige adipocytes. UCP1 is an inner-membrane mitochondrial protein for both BAT and beige adipocytes, and its activation causes a reflux of protons into the mitochondrial matrix, which bypasses ATP synthase and induces heat dissipation [18]. Thus, the increased UCP1 content further confirmed the stimulatory effect of SBP on the browning of white adipose tissue in lambs. Quercetin is a polyphenolic flavonoid that is present in sea buckthorn, and has been proven to upregulate UCP1 expression in the browning of 3T3-L1 adipocytes [19]. Therefore, the quercetin in SBP might contribute to the appearance of beige fat cells in iWAT in lambs.

In mammals, white adipocytes possess a single fat droplet and low mitochondrial density, whereas beige adipocytes have several small fat droplets and a greater number of mitochondria. In this trial, dietary SBP increased mitochondrial numbers in sheep iWAT in a dose-dependent manner. PGC-1α is a transcriptional coactivator that plays a central role in mitochondrial biogenesis, and its expression is highly inducible by physiological cues and biological components [20]. The effect of SBP on the expression of PGC-1α was consistent with a previous study in murine skeletal muscle, showing that polyphenolic flavonoids increase PGC-1α content [21]. PGC-1α activates the transcription of mitochondrial *UCP1* in BAT through interactions with PPARγ and the thyroid receptor [22], which might explain the observed *UCP1* expression pattern in the present study. Moreover, PGC-1α increases the expression of the nuclear respiratory factors (NRFs), which can further regulate mitochondrion-related gene expression, and enhance the transcriptional activity of NRF-1 on its target genes [23]. Collectively, these results suggest that dietary SBP might regulate PGC-1α content to drive mitochondrial biogenesis in the iWAT of lambs.

PRDM16, one of the most important transcriptional coregulators of brown/beige adipocyte differentiation, was upregulated in the 16SBP group. Indeed, PRDM16 and C/EBPβ synergistically stimulate *PGC-1α* transcriptional activity [24]. Moreover, overexpression of C/EBPβ alone is sufficient to induce a brown fat cell-like phenotype in white adipocytes [25]. As expected, C/EBPβ was dramatically induced in the 16SBP lambs. Therefore, these data suggested that dietary SBP acted on various molecular targets regulating the browning of iWAT in lambs. A previous study showed that irisin stimulates the browning of white adipocytes [26]; although quercetin administration to hyperbaric hypoxic rats increases irisin content [27], dietary SBP supplementation in lambs did not alter irisin content in their iWAT.

The presence and activity of beige adipocytes are associated with improved insulin sensitivity [28]; therefore, the induction of beige adipocytes by SBP suggested its potential to increase insulin sensitivity in lamb iWAT. Glucose uptake is dependent on glucose transporters, and GLUT4 is the major transporter. Our data suggested that dietary SBP supplementation increased both mRNA and protein contents of Glut4. Insulin signaling pathways play an important role in regulating translocation of Glut4 proteins from the cytosol to the plasma membrane. The binding of insulin to its receptor causes phosphorylation and activation of IRS1, which further phosphorylates PI3K and Akt, leading to Glut4 translocation [29]. Here, 16% dietary SBP supplementation increased phosphorylation of both IRS1 and Akt, in line with a recent study showing that palmitoleic acid extracted from sea buckthorn alleviates insulin resistance through the Akt signaling pathway [30].

To further pursue possible mechanisms by which dietary SBP may participate in the regulation of iWAT browning, we analyzed signal transduction pathways that might be involved in browning by western blotting. Previous studies proved that flavonoids from sea buckthorn inhibited p38 activity in macrophages [17], and inhibition of the p38 signaling pathway promoted white adipose tissue browning [31]; thus, we analyzed phospho-p38 protein content. Unexpectedly, no change of phospho-p38 was observed in the SBP feeding lambs, indicating that p38 signaling might not be involved in the browning of inguinal white adipose tissue induced by dietary SBP. AMPK is a cellular energy sensor that has recently been demonstrated to be important in the development and maintenance of functional beige adipose tissue [32]. Moreover, a previous study demonstrated that flavonoids activate AMPK in 3T3-L1 preadipocytes [33]. Once activated, AMPK directly affects PGC-1α activity through phosphorylation [34], which further regulates mitochondrial biogenesis. Thus, the activation of AMPK by dietary SBP supplementation in the present study might provide an alternative mechanism by which dietary SBP promotes browning of iWAT in lambs.

## 5. Conclusions

Dietary SBP supplementation in lambs effectively promoted iWAT browning and mitochondrial biogenesis, which might be through activation of the AMPK–PGC-1α–UCP1 signaling pathway. Additionally, dietary SBP supplementation upregulated Glut4 expression and increased insulin sensitivity in inguinal white adipose tissue. These results might provide insight for modulating heat production in young lambs through nutrition regulation.

## Figures and Tables

**Table 1:** Composition and nutrient levels of diets.

Dietary Ingredient	Control	7.8SBP	16SBP
Corn, %	28.90	24.20	23.00
Soybean meal, %, 44% crude protein	13.40	12.30	12.30
Wheat bran, %	3.00	3.00	3.00
Wheat shorts, %	5.00	5.00	5.00
Oil cake of flax seed, %	4.70	4.70	4.70
Mineral/vitamin premix, %	4.00	4.00	4.00
SBP, %	0	7.80	16.00
Naked oats straw, %	25.00	27.00	20.00
Potato rattan, %	15.00	11.00	11.00
Limestone, %	0.50	0.50	0.50
Sodium Chloride, %	0.50	0.50	0.50
Total	100.00	100.00	100.00
Nutritional level			
Gross Energy (MJ/kg)	17.70	17.70	17.50
Crude protein, (%)	13.20	13.20	13.20
Ether extract, EE, (%)	1.09	2.29	2.60
Neutral detergent fiber, NDF, (%)	44.10	41.90	40.50
Acid detergent fiber, ADF, (%)	28.40	28	26.80
Calcium, (%)	0.81	0.75	0.81
Phosphorus, (%)	0.64	0.63	0.58
Flavonoids, (%)	0.41	0.69	1.02

**Table 2:** Primer sequences for real-time PCR.

Name	Sequence (5’–3’)	Accession Number
*UCP1*	TTGCTTCTCTCAGGATCGGC GTGGGTTGCCCAATGAACAC	NM_001280694.1
*PRDM16*	GCCTGTTTCTCTTCTGTCCCC GCCAACAGGACGGTGTTATTT	XM_012187950.2
*Cidea*	TGCATCCTCCAAGCGTTTCT CCTCCTGTTCAGTCCACACC	XM_012103597.
*COX7A1*	CGGTGCAACAGACAACATCC GTCCCGCAGACTTCTTGGTT	XM_012120571.2
*PGC-1α*	TGTCGGATGCTTGCTTGAGT TACGGTTGTAACGCAGGACCT	XM_015106709.1
*TFAM*	AAGGCGCTGCAGGGAAG CGCAAAACTAAAGGGGGAGC	XM_015104510.1
*NRF-1*	AGCCGCTCTGAGTGGATCT AACATGCTGTGCTCGGTGTA	AY368269
*D-Loop*	GCATAGGACTAGGGCTTAGCTT GGAGATTGGTGGTGTGGCATA	KY662382.1
*GAPDH*	ACAGTCAAGGCAGAGAACGG CCAGCATCACCCCACTTGAT	NM_001190390.1
*RPL13*	GCAAAAAGGGCCAAGGAAGC CAAAGGTCAGACACACCCCA	XM_015100414.1
